# Iron Deficiency Anemia-Induced Neutropenia in Adult Female

**DOI:** 10.7759/cureus.8899

**Published:** 2020-06-29

**Authors:** Elabbass Abdelmahmuod, Mohamed A Yassin, Mohanad Ahmed

**Affiliations:** 1 Internal Medicine, Hamad Medical Corporation, Doha, QAT; 2 Hematology and Oncology, Hamad General Hospital, Doha, QAT

**Keywords:** ida, neutropenia, anemia

## Abstract

Iron deficiency anemia is a common cause of anemia that develops when body stores of an iron drop too low to support normal red blood cell (RBC) production. Inadequate dietary iron, impaired iron absorption, bleeding, or loss of body iron in the urine may be the cause. Iron is a key part of red blood cells. Without iron, the blood cannot carry oxygen effectively. Our body normally gets iron through the diet. It also reuses iron from old red blood cells. A little is known about the association between iron deficiency anemia and neutropenia. Here we report a 44-year-old female who presented with iron deficiency anemia and found to have neutropenia recovered after she received intravenous (IV) iron therapy. However, she did not develop any serious infections during the neutropenia and responded to iron therapy.

## Introduction

Iron deficiency anemia occurs when iron deficiency is severe enough to diminish erythropoiesis and cause the development of anemia. Iron equilibrium in the body normally is regulated carefully to ensure that sufficient iron is absorbed to compensate for body losses of iron [1].

Symptoms of iron-deficiency anemia often take a long time to develop. People may not know they have it until the symptoms are severe. However, a person with an iron deficiency can have some of the following symptoms: general weakness, dizziness or lightheadedness, extreme fatigue, palpitation, easily broken and brittle nails pall, chest pain, shortness of breath, headaches, soreness or inflammation of the tongue, cravings for non-nutritive things such as dirt, starch, and poor appetite especially in children [2].

The diagnosis of iron deficiency anemia (IDA) confirmed by one of the following findings in the appropriate clinical setting: Serum ferritin <30 ng/mL, transferrin saturation <19%, mostly used in patients for whom the ferritin is thought to be unreliable due to an inflammatory state, anemia that resolves upon iron administration or absence of stainable iron in the bone marrow (providing that adequate staining controls are performed) [3, 4].

Neutropenia refers to the abnormal reduction in the number of circulating neutrophils. It occurs when there is a reduction in the number of produced neutrophils or increased destruction or both. Infection, drugs, malignancy, megaloblastosis, hypersplenism, and immune-neutropenia are known causes of leukopenia [5].

IDA presents with thrombocytosis, however, there is a rare association between IDA and neutropenia. Here we would like to shed the light on this rare presentation. The primary threat when dealing with neutropenia lies in the increased risk of infection.

To the best of our knowledge, this is the second important case in the literature that discusses the relationship between iron deficiency and neutropenia. The first case, published in 2014, was of elderly female known to have iron deficiency anemia and persistent unexplained chronic leukopenia [6].

## Case presentation

A 44-year-old previously healthy female, who presented with generalized body weakness, was found to have severe anemia (hemoglobin 5.7 gm/dl; normal value is 12-15 gm/dl). She denied other symptoms. On examination, she showed a severe pallor and no hepatosplenomegaly (Tables 1-3).

**Table 1:** Complete blood count (CBC) Complete blood count was performed in General Hematology on 05/03/2020. WBC: White blood cell; RBC: Red blood cell; Hgb: Hemoglobin; Hct: Hematocrit; MCV: Mean corpuscular volume; MCH: Mean corpuscular hemoglobin.

Detail	Value	Flags	Normal Range
WBC	3.8 x 10^3^/uL	LOW	4.0-10.0
RBC	3.2 x 10^6^/uL	LOW	3.8-4.8
Hgb	5.7 gm/dL	CRIT	12.0-15.0
Hct	20.3%	LOW	36.0-46.0
MCV	64.0 fL	LOW	83.0-101.0
MCH	18.1 pg	LOW	27.0-32.0
Platelet	474 x 10^3^/uL	HI	150-400
Absolute neutrophil count Auto# (ANC)	1.86 x 10^3^/uL	LOW	2.00-7.00
Lymphocyte Auto	1.32 x 10^3^/uL		1.00-3.00

**Table 2:** Iron profile >> Severe iron deficiency anemia Iron profile was performed on Blood Chemistry on 05/03/2020. TIBC: Total iron binding capacity

Detail	Value	Flags	Normal Range
Iron	5.1 umol/L	LOW	5.90-18.30
TIBC	70 umol/L		45-80
Fe% Saturation	8%	LOW	15-45
Ferritin	3 mcg/L	LOW	6.0-44.0

**Table 3:** B12, folate and thyroid function test TSH: Thyroid-stimulating hormone; RBC Fol: Red blood cell folate.

Detail	Value	Normal Range
TSH	1.68 mIU/L	0.30-4.20
FT4	15.1 pmol/L	11.6-21.9
Vit B12	349.0 pmol/L	145.0-596.0
RBC Fol	1,605 nmol/L	1,187-2,854

The patient received one dose of iron ferric carboxymaltose (Ferinject) 1000 mg. Her symptoms, anemia, and neutropenia improved after therapy (Table 4).

**Table 4:** Labs after iron therapy WBC: White blood cell; RBC: Red blood cell; Hgb: Hemoglobin; Hct: Hematocrit; MCV: Mean corpuscular volume.

Detail	Value	Normal Range
WBC	6.3 x 10^3^/uL	4.0-10.0
RBC	3.4 x 10^6^/uL	3.8-4.8
Hgb	6.1 gm/dL	12.0-15.0
Hct	24.3%	36.0-46.0
MCV	72.3 fL	83.0-101.0
Platelet	529 x 10^3^/uL	150-400
Absolute Neutrophil count Auto# (ANC)	4.3 x 10^3^/uL	2.0-7.0
Lymphocyte Auto #	1.1 x 10^3^/uL	1.0-3.0

## Discussion

Iron deficiency anemia affects both mental and physical development. It is the most common micronutrient deficiency worldwide. It is commonly occurred in developing countries due to inadequate nutrition. Neutropenia is defined as abnormal reduction in neutrophil concentration (an absolute neutrophil count (ANC) <1,500 cells/lL). Neutropenia can be due to primary causes such as congenital neutropenia or secondary causes such as B12 and folate deficiencies or bone marrow depression due to chemotherapy, and very common but transiently due to self-limiting upper respiratory tract virus diseases [7].

Our patient was suffering from iron deficiency anemia due to low hemoglobin (Hgb), low mean corpuscular volume (MCV), low ferritin, high total iron binding capacity (TIBC), and low transferrin saturation. Other etiologies of microcytic hypochromic anemia such as thalassemia were excluded by the other tests such as normal Hgb electrophoresis. She also suffered from neutropenia indicated by low ANC (<1,500 cells/lL); vitamin B12 and folate deficiency were ruled out as a cause for neutropenia because of the normal serum levels. Forty percent of children who suffered from iron deficiency anemia, also suffered from infections. Some cytokines are contributed in immune activation, response to infection as well as iron transport and metabolism. Increased risks of infections have been noticed in some IDA patients but the etiology is not well-known [6].

IDA affects thyroid metabolism in animals and humans and negatively affects the growth and development of children. Hypothyroidism is associated with anemia and adding iron to thyroxine therapy improves hypothyroidism compared to thyroxine therapy alone [8]. It appears that all forms of chronic anemia hurt linear growth during all stages of growth (infancy, childhood, and adolescence). Infants with chronic IDA may have delayed cognitive, motor, and affection [9]. The effects on male fertility were explored by Soliman et al. in their study which showed that correction of IDA with intravenous iron therapy (IVI) is associated with significant enhancement of sperm parameters and increased concentrations of serum luteinizing hormone (LH), follicle stimulating hormone (FSH), and testosterone. These effects on spermatogenesis are reached by an unknown mechanism and suggest several pathways that need further human and/or experimental studies [10]. In another study looking for the effects of IDA on glucose metabolism, Soliman et al. suggest that among non-diabetic and diabetic individuals IDA is associated with higher concentrations of hemoglobin A1c (HbA1c). Iron replacement therapy decreases HbA1c in both diabetic and non-diabetic individuals. This implies that the iron states must be considered during the interpretation of HbA1c concentrations in diabetic or non-diabetic patients. Early diagnosis and treatment of ID in diabetic patients can improve their glycemic control and may prevent or delay complications [11,12].

## Conclusions

There is a lack of clinical research regarding the effect of iron deficiency anemia on white blood cells (WBC). As our case suggests, some patients diagnosed with neutropenia who clinically present as having unexplained neutropenia might be suffering from iron deficiency anemia-induced neutropenia. Our findings also propose iron supplement therapy as a possible treatment for this form of neutropenia.

Clinicians should consider the possibility that neutropenia may be caused by iron deficiency, and should pay attention to such presentation as this can lead to infection because of neutropenia.
